# Supplementary material for: The evolution and biological correlates of hand preferences in anthropoid primates
Source: eLife. 2022 Dec 1;11:e77875. doi: 10.7554/eLife.77875 (PMC9714969; doi:10.7554/eLife.77875)

Supplementary File 1: Phylogenetic Bayesian regression models on individual lateralization parameters

Abbreviations:

Est. Error: Estimate error

l-95% CI: lower limit of 95% credible interval

u-95% CI: upper limit of 95% credible interval

Rhat: Potential scale reduction factor

Bulk_ESS: bulk effective sample size

Tail_ESS: tail effective sample size

Notable effects were assumed when the model's 95% credible intervals of intercept and respective regression coefficients did not overlap with zero (marked in bold).

Figures visualize estimates for individual predictors on the left, and the number of chains and performed iterations on the right.

Model 1 (Lateralization direction, total sample)

Group-level effects							
	Estimate	Est. Error	l-95% CI	u-95% CI	Rhat	Bulk_ESS	Tail_ESS
sd(Intercept)	0.02	0.01	0.01	0.04	1.00	1376	2057
Population-level effects							
Intercept	-0.02	0.10	-0.22	0.17	1.00	1530	1565
Sex:male	0.00	0.03	-0.06	0.06	1.00	5086	2613
Age:subadult	-0.07	0.04	-0.15	0.00	1.00	5838	3386

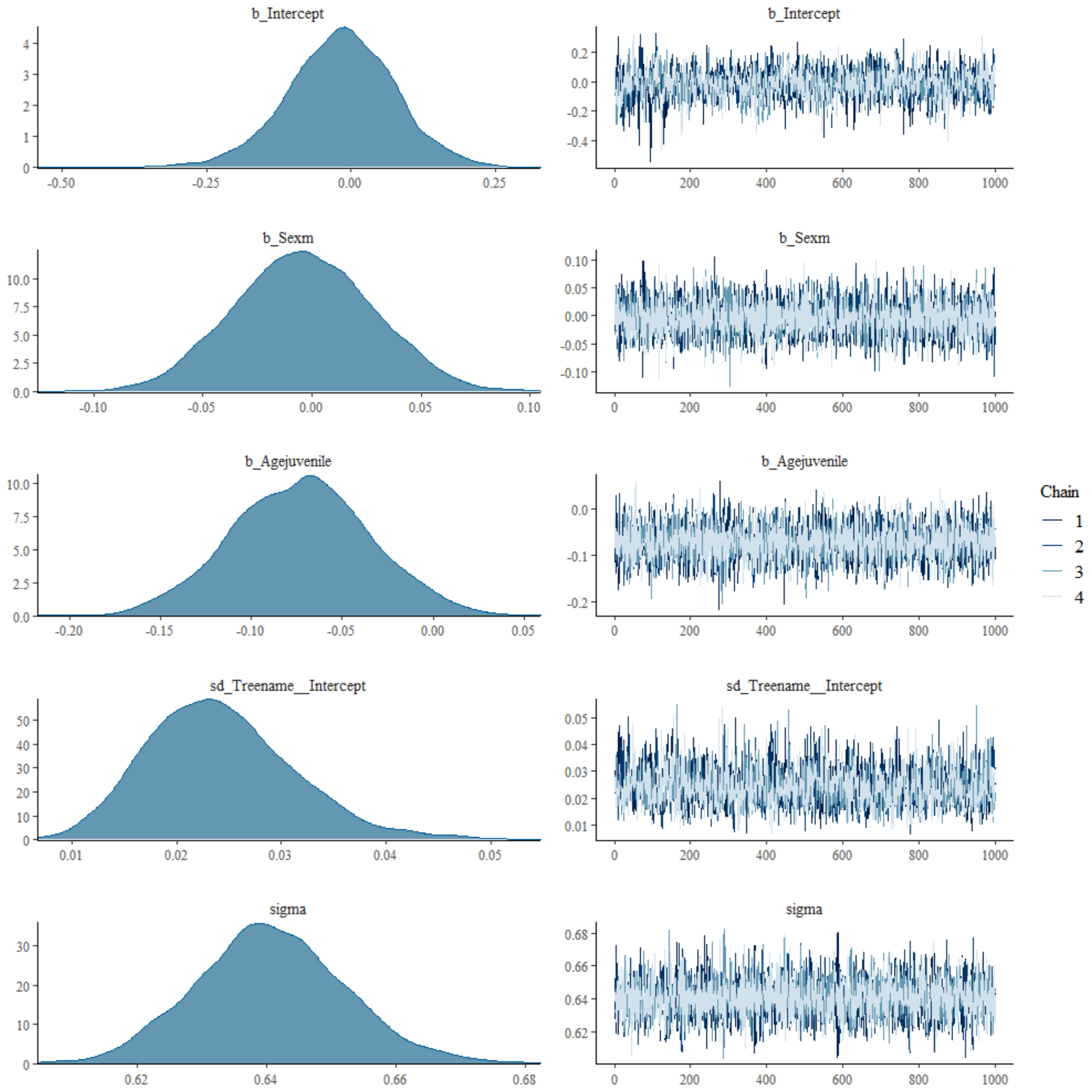

Model 2 (Lateralization strength, total sample)

Group-level effects							
	Estimate	Est. Error	l-95% CI	u-95% CI	Rhat	Bulk_ESS	Tail_ESS
sd(Intercept)	0.04	0.01	0.03	0.05	1.00	1149	1835
Population-level effects							
Intercept	0.67	0.14	0.40	0.93	1.00	829	1754
Sex:male	0.02	0.02	-0.01	0.05	1.00	4560	3068
**Age:subadult**	**-0.08**	**0.02**	**-0.11**	**-0.05**	**1.00**	**4581**	**3076**

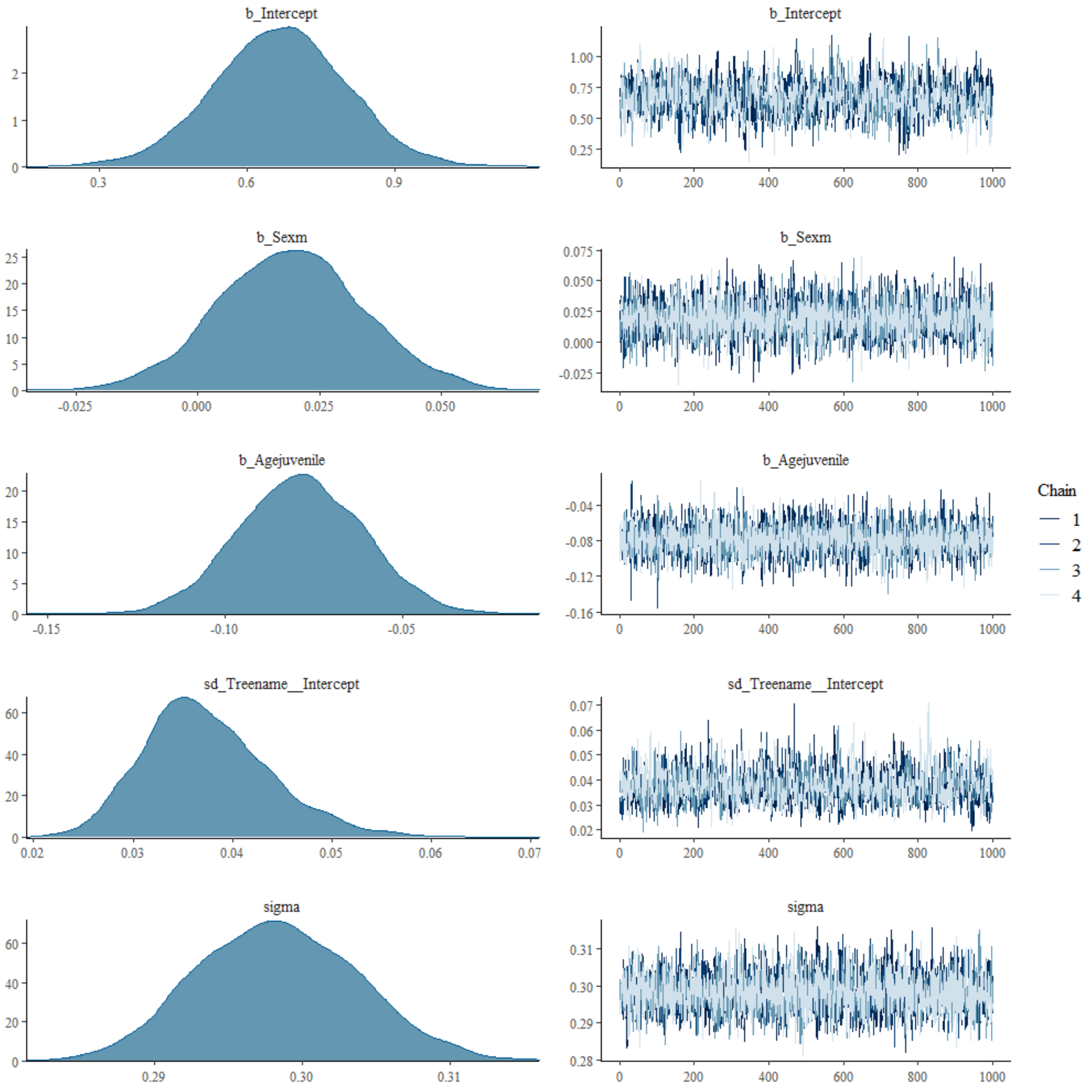

Model 3 (Lateralization direction, Platyrrhini)

Group-level effects							
	Estimate	Est. Error	l-95% CI	u-95% CI	Rhat	Bulk_ESS	Tail_ESS
sd(Intercept)	0.02	0.02	0	0.08	1.00	1717	784
Population-level effects							
Intercept	0.05	0.16	-0.28	0.38	1.01	1005	615
Sex:male	-0.11	0.09	-0.30	0.07	1.00	6417	6563
Age:subadult	-0.13	0.14	-0.41	0.15	1.00	9115	7120

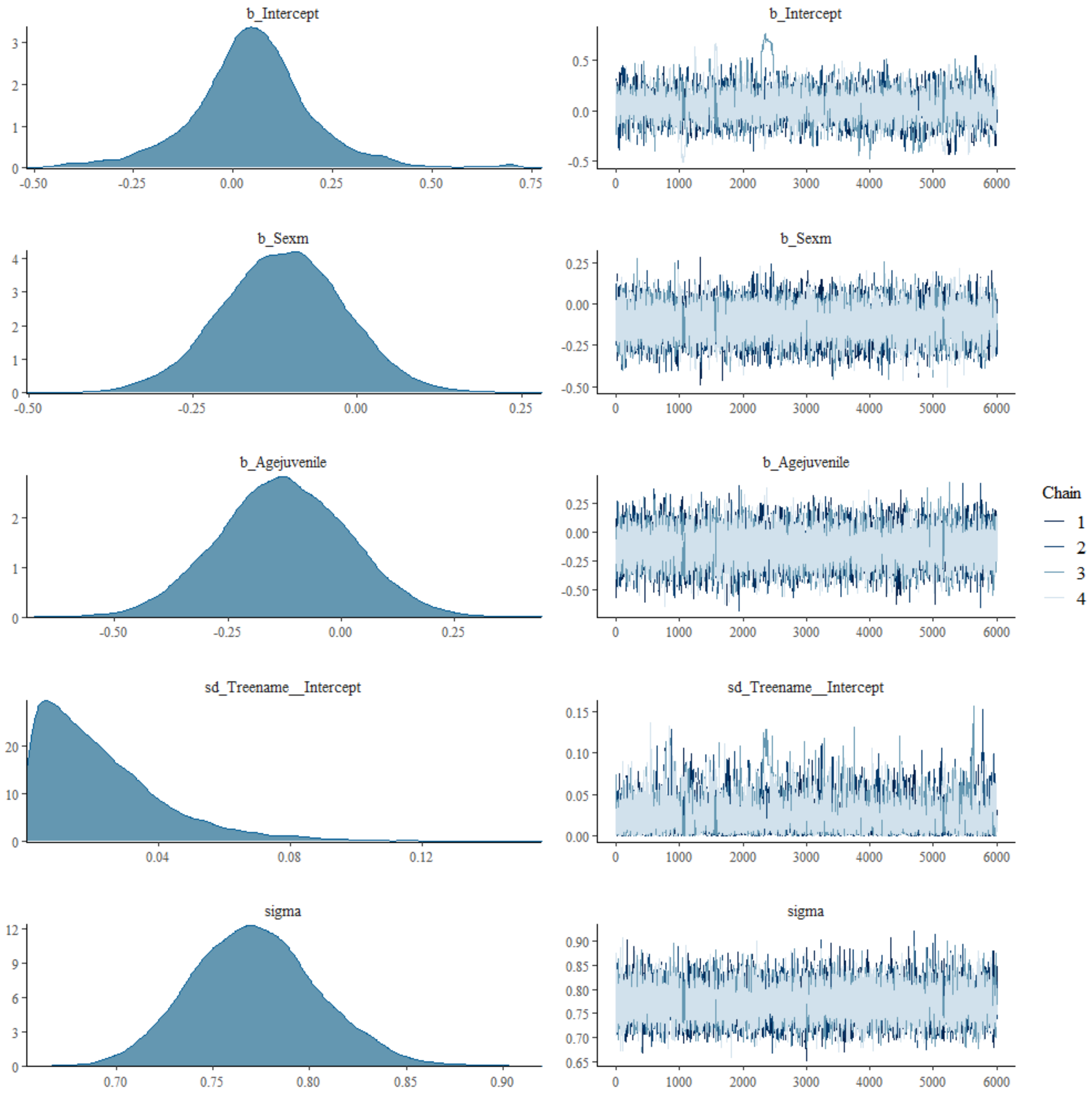

Model 4 (Lateralization strength, Platyrrhini)

Group-level effects							
	Estimate	Est. Error	l-95% CI	u-95% CI	Rhat	Bulk_ESS	Tail_ESS
sd(Intercept)	0.04	0.02	0.02	0.08	1.00	1692	1048
Population-level effects							
Intercept	0.76	0.23	0.29	1.21	1.00	1854	877
Sex:male	-0.05	0.04	-0.12	0.02	1.00	13737	6729
Age:subadult	-0.03	0.05	-0.14	0.07	1.00	7848	2185

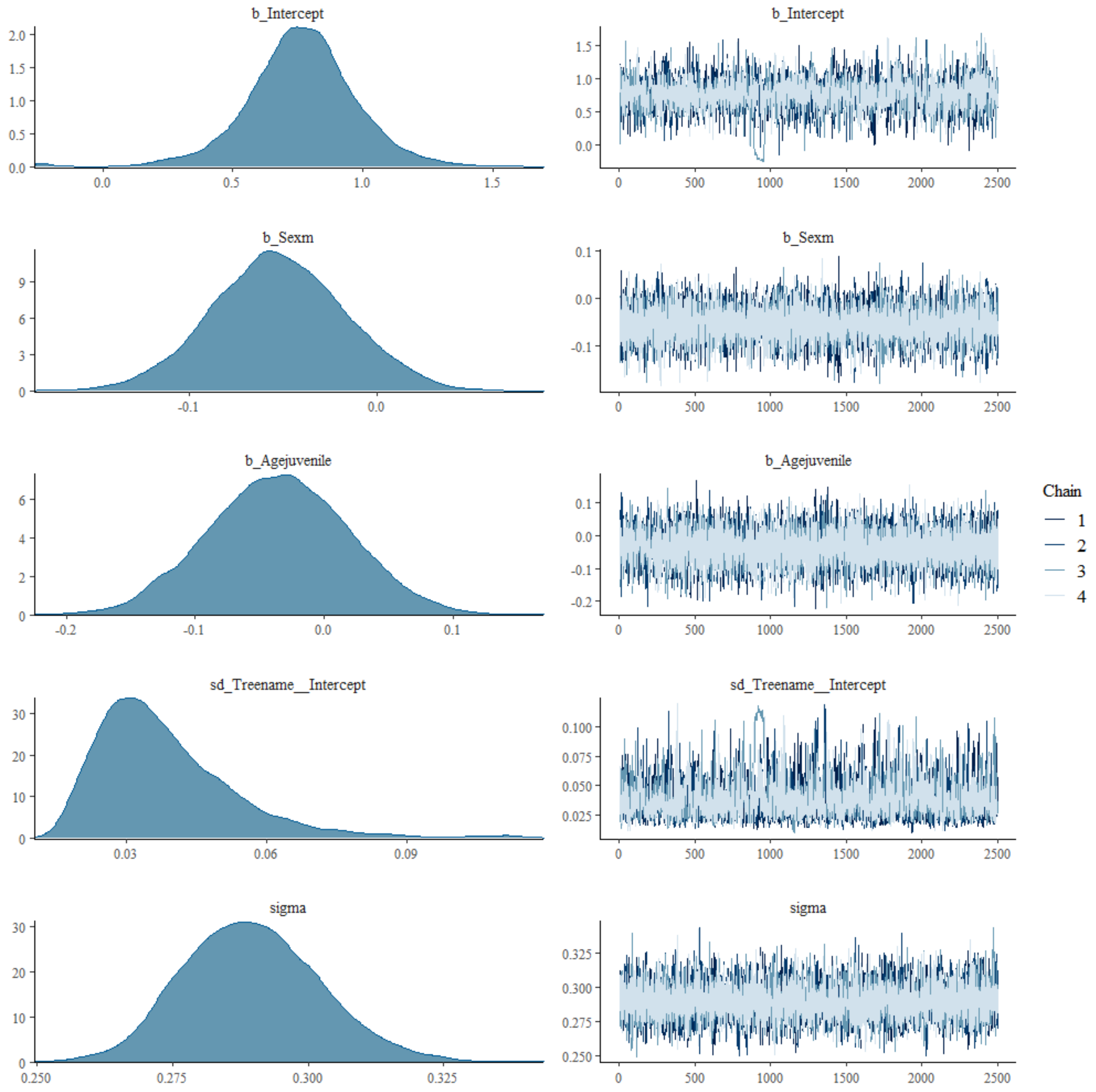

Model 5 (Lateralization direction, Cercopithecoidea)

Group-level effects							
	Estimate	Est. Error	l-95% CI	u-95% CI	Rhat	Bulk_ESS	Tail_ESS
sd(Intercept)	0.02	0.01	0.09	0.05	1.01	640	419
Population-level effects							
Intercept	-0.06	0.17	-0.48	0.27	1.01	541	194
Sex:male	0.02	0.06	-0.10	0.14	1.00	3062	1473
Age:subadult	-0.08	0.09	-0.25	0.09	1.00	3388	2618

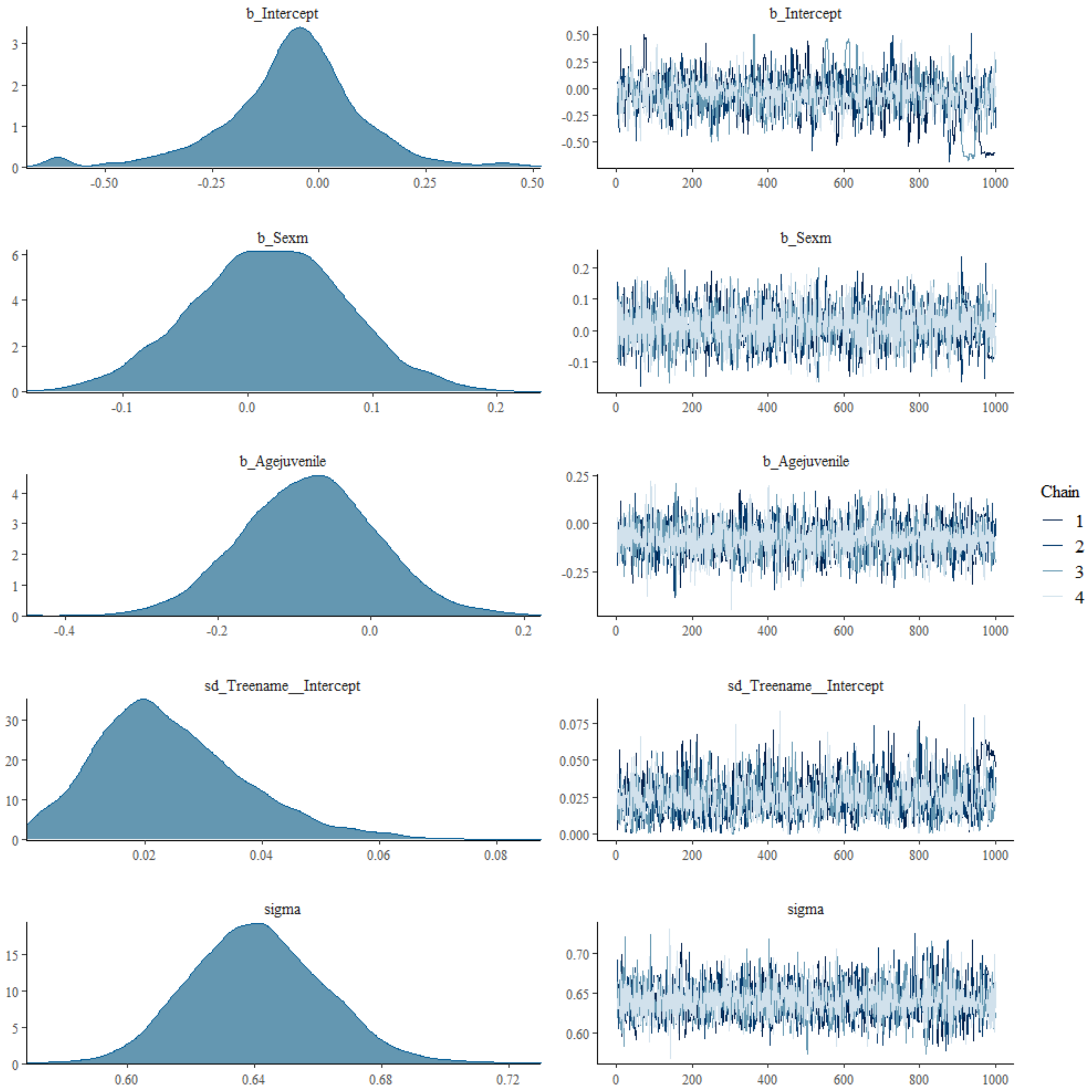

Model 6 (Lateralization strength, Cercopithecoidea)

Group-level effects							
	Estimate	Est. Error	l-95% CI	u-95% CI	Rhat	Bulk_ESS	Tail_ESS
sd(Intercept)	0.05	0.01	0.03	0.08	1.01	772	1284
Population-level effects							
Intercept	0.60	0.30	0	1.20	1.00	1060	1406
Sex:male	0.05	0.03	0	0.11	1.00	3981	2986
Age:subadult	-0.04	0.04	-0.12	0.04	1.00	4070	3171

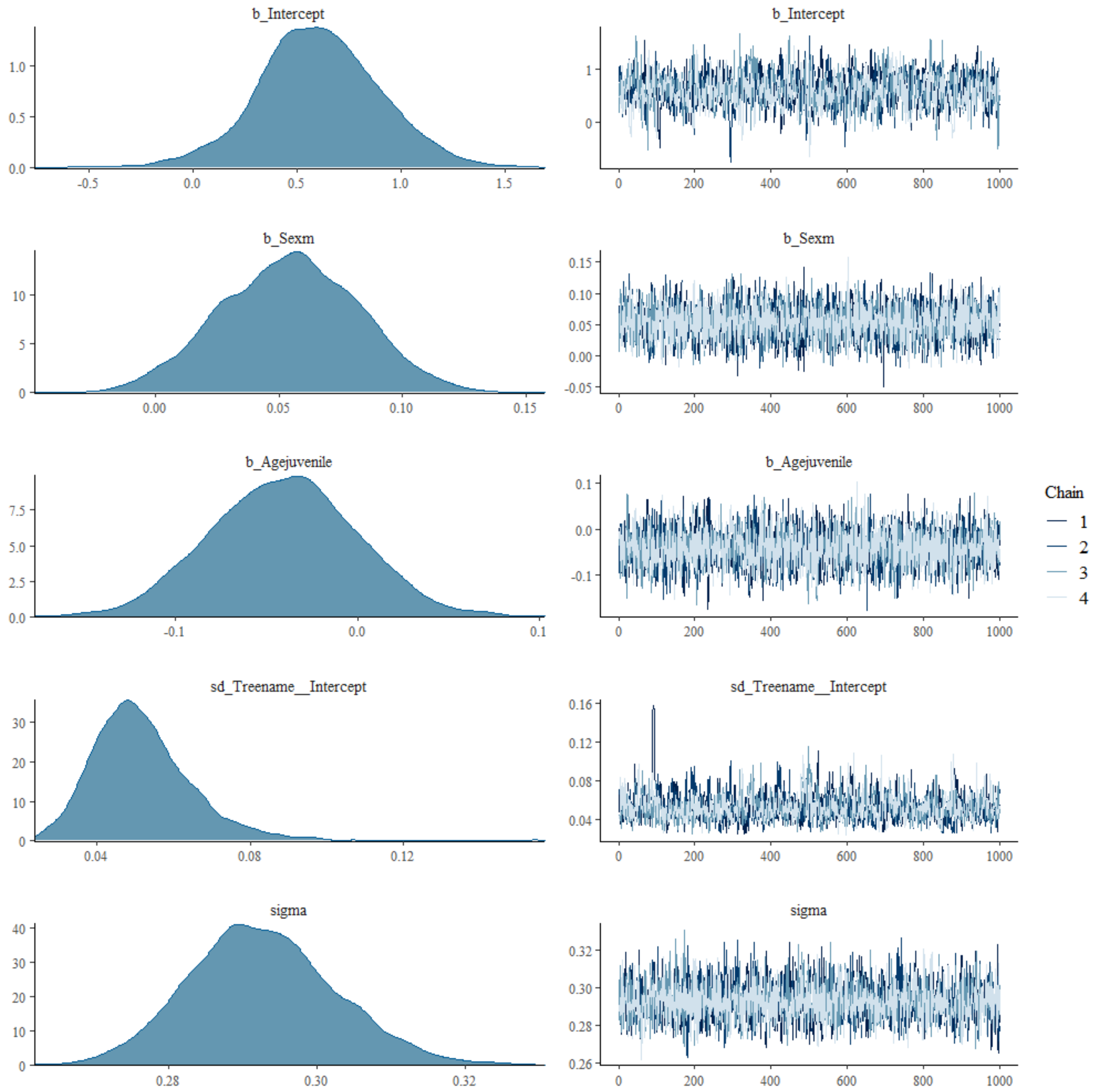

Model 7 (Lateralization direction, Hominoidea)

Group-level effects							
	Estimate	Est. Error	l-95% CI	u-95% CI	Rhat	Bulk_ESS	Tail_ESS
sd(Intercept)	0.05	0.02	0.02	0.10	1.00	1862	908
Population-level effects							
Intercept	0.04	0.30	-0.55	0.70	1.00	1806	850
Sex:male	0.04	0.07	-0.09	0.17	1.00	9879	7468
**Age:subadult**	**-0.15**	**0.07**	**-0.28**	**-0.01**	**1.00**	**8675**	**7153**

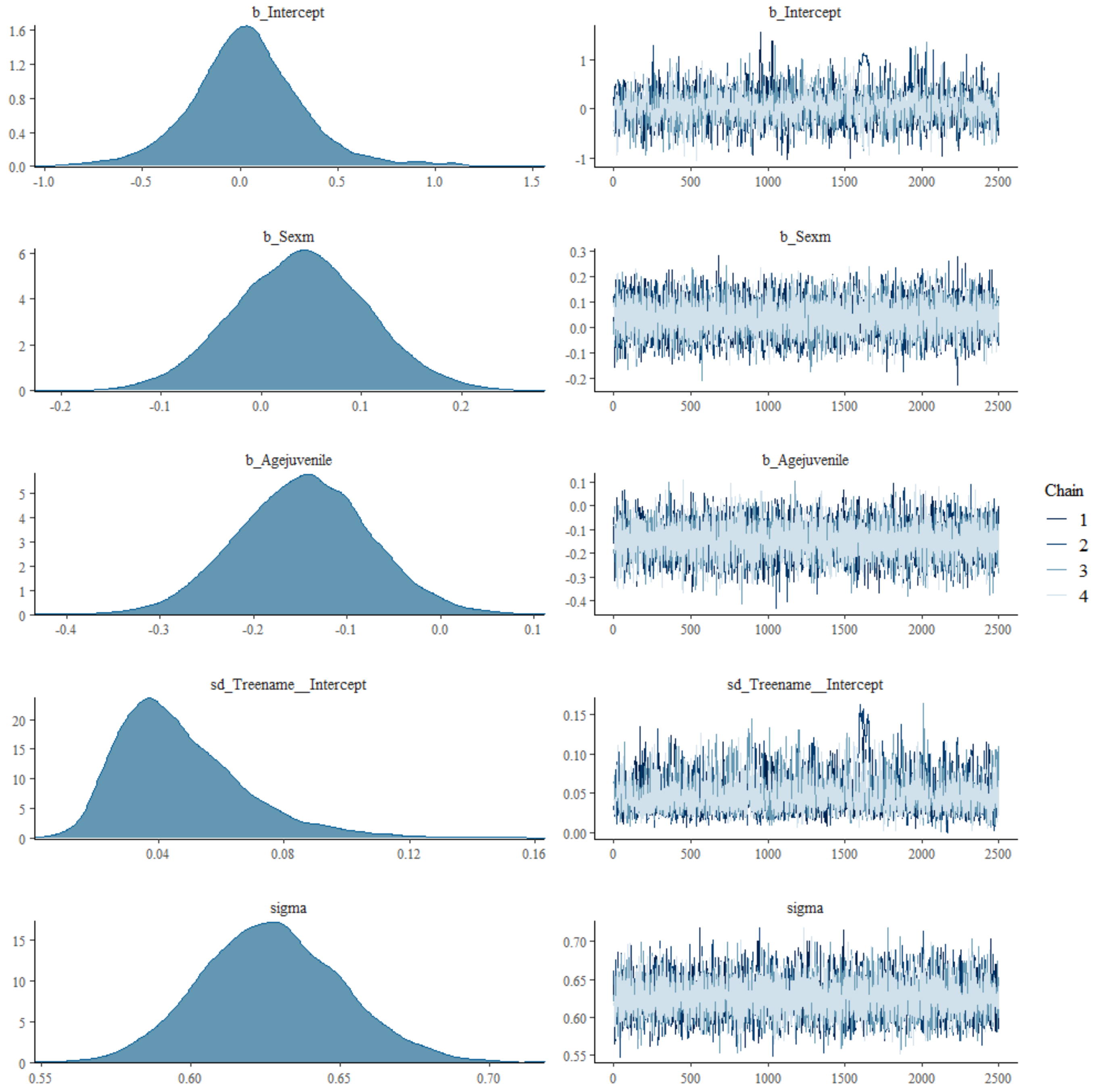

Model 8 (Lateralization strength, Hominoidea)

Group-level effects							
	Estimate	Est. Error	l-95% CI	u-95% CI	Rhat	Bulk_ESS	Tail_ESS
sd(Intercept)	0.03	0.02	0.00	0.07	1.00	1082	903
Population-level effects							
Intercept	0.56	0.16	0.17	0.88	1.01	754	240
Sex:male	0.03	0.03	-0.03	0.10	1.00	5377	8507
**Age:subadult**	**-0.09**	**0.04**	**-0.16**	**-0.02**	**1.00**	**3684**	**5352**

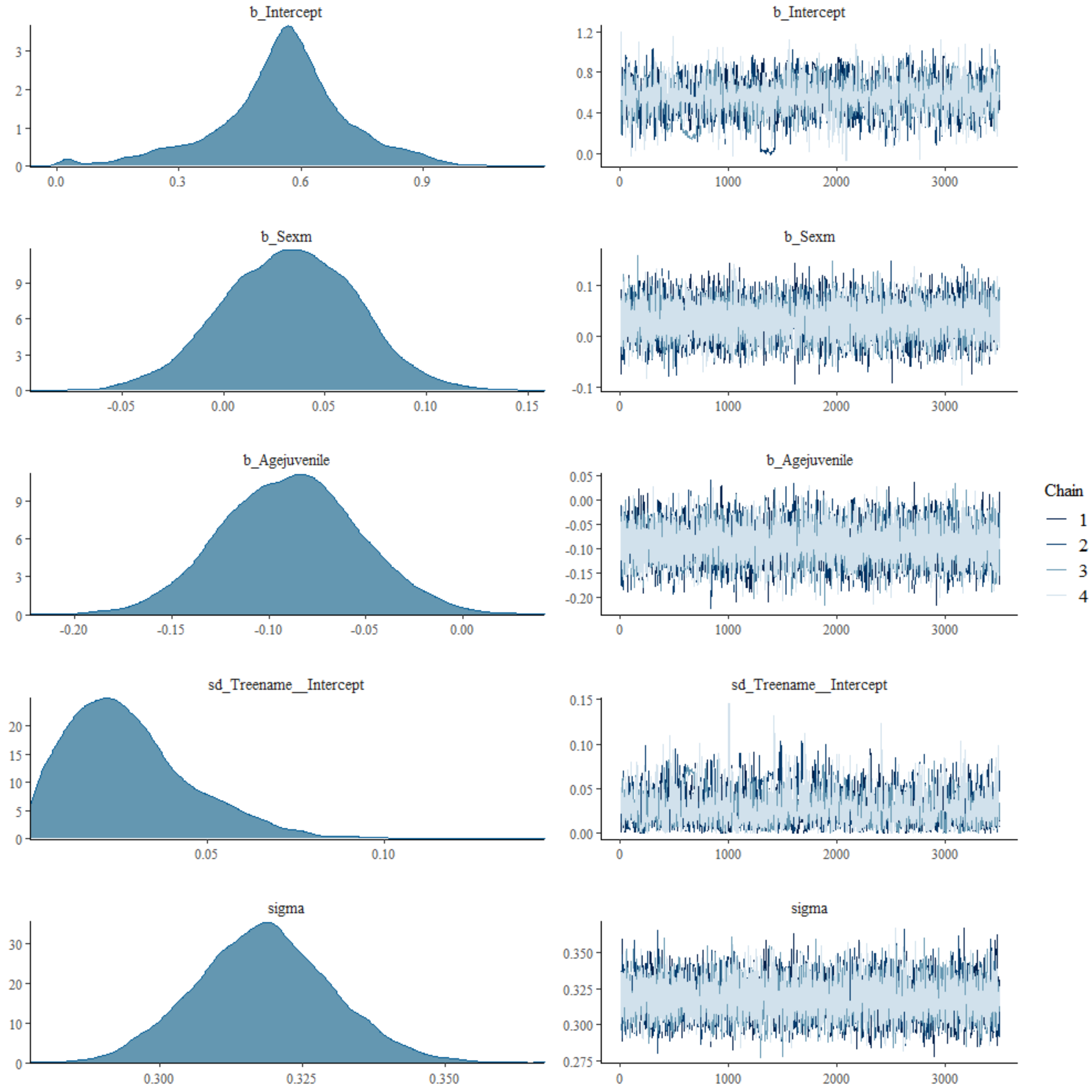

Supplement: Supplementary file 2. [file elife-77875-supp2.pdf]
